# Bereavement management during COVID-19 Pandemic: One size may not fit all!

**DOI:** 10.7189/jogh.11.03009

**Published:** 2021-01-16

**Authors:** Ashwin Rammohan, Priya Ramachandran, Mohamed Rela

**Affiliations:** 1 The Institute of Liver Disease & Transplantation, Dr. Rela Institute & Medical Centre, Bharath Institute of Higher Education & Research, Chennai, India; 2 The Ray of Light Foundation, Kanchi Kamakoti CHILDS Trust Hospital, CHILDS Trust Medical Research Foundation, Chennai, India

The way in which individuals react to the death of a loved one varies across cultures. The coping mechanisms to this grief however, remain remarkably constant. Irrespective of race, religion or culture death is almost universally followed by a funeral service or ritual [1]. Across the globe, communities have developed these rites to enable individuals and families cope and deal with the loss of their loved one. A funeral ritual allows for a culturally accepted expression of emotions, emphasizing the irreversibility of death. It also initiates the recovery processes of continuity, transition and transformation [2].

Deaths during communicable disease epidemics are even more distressing as they defy the concepts of an “ideal death”. Family members may not have the opportunity to achieve closure by resolving “unfinished business”. The pain and guilt of not physically seeing or being with their loved ones during their suffering exacerbates the grief. Furthermore, a lack of social recognition with impaired support system along with the absence of last rites results in a state of “disenfranchised grief”. This is likely to result in a prolonged grief disorder, a condition which imperils the physical and psychological well-being of an individual [1,3].

The Ebola and Marburg virus disease epidemics were examples where strict regulations were in place for the management of the deceased and performance of last rites [4]. The rationale behind such strict guidelines were among others factors, a high case fatality rate observed in those who had prolonged contact with the corpse. Funerals also posed a substantial risk for transmission due to other reasons. First, an increased viral load was noted in the non-survivors more so during the terminal stages of the disease and this intuitively translated to a higher risk of transmission. Second, traditional practices across cultures included close contact with the body including washing and other preparations for public display. This meant that there was a prolonged period of contact of individuals with the corpse. Lastly, it is customary for these last rites to be well attended by family, friends and people within the community. Attendance was usually important to demonstrate deference and to establish socio-political and financial rights [1]. These attendees could then establish new chains of transmission, leading to an unchecked transmission of the virus.

While statistics on the number of deceased are readily available during an epidemic, the grief of a loved one remains intangible. Moreover, with the COVID-19 pandemic continuing unabated and due to the purported contagious nature of the virus, hospital rules prohibit family’s access to the deceased [3,5]. For those dying in overwhelmed facilities, bodies may not be treated with the dignity they would ordinarily receive. With beleaguered staff quickly making beds available for new patients, dead bodies may end up being piled in corridors or trucks and disposed of without the family getting to see them. The WHO and Governments have placed restrictions on performance of last rites based on previous experience with highly communicable fatal diseases [6,7]. Main highlights of the guidelines include rules for removal of the body from the isolation area, handling of dead body in the mortuary and at the crematorium/burial ground. There is also a wide variation in the actual enforcement of these regulations. In the majority of cases, there is a tendency towards a much stricter enforcement of isolation procedures denying the relatives any form of closure. Some countries have laid down stringent guidelines which extend beyond the simple concept of controlling the spread of infection. For example, the Srilankan Government dictates that all COVID-19 deaths be compulsorily cremated [8]. Online memorial services, virtual support groups, telephonic grief counselling and other innovations may provide short-term bereavement support for survivors of COVID-19 deaths [3,5]. While pragmatic, these may not be the most durable long-term models of helping people cope with bereavement. Moreover, the opportunity to respect cultures, traditions and the need to provide comfort to those left behind remain incomplete.

**Figure:**
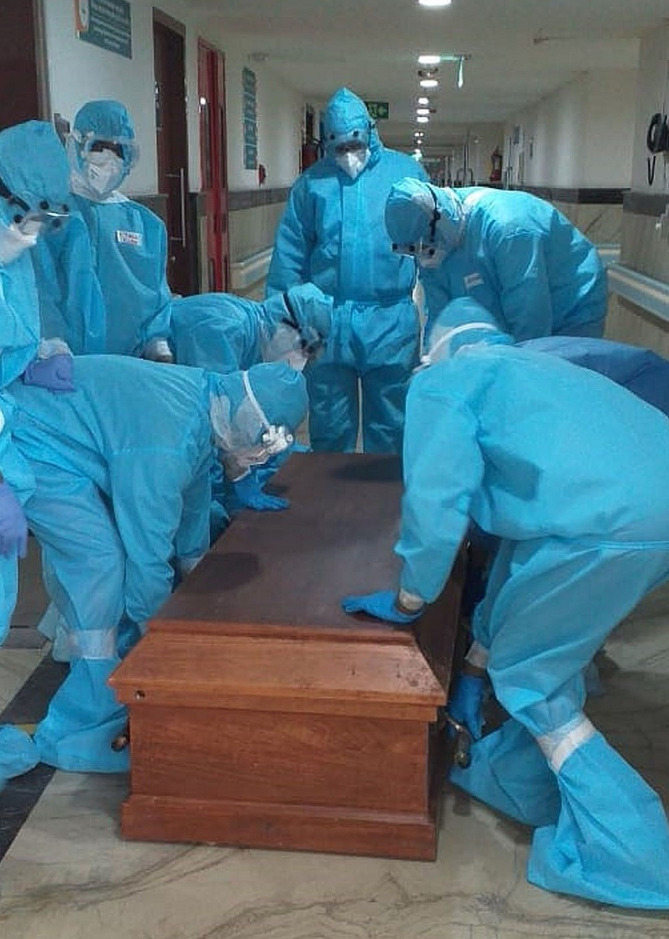
Photo: Hospital authorities in protective suits follow strict precautions in the management of the mortal remains of a COVID-19 patient (from the author’s collection, used with permission).

With the pandemic raging on especially in the developing world, it is important to reassess the infectivity quotient of COVID-19 among the deceased. There are no models or real-life situations to objectively define this metric. Post-mortem studies have shown only autopsied lung tissue and any aerosolised bodily fluid to contain the infective particles [6]. Currently, regulations based on previous experiences with other highly communicable diseases like Ebola and Marburg virus have been used to define the radical and stringent means of deceased disposal. In this context, there exists important differences between COVID-19 deaths and other communicable diseases which need highlighting. Most of the COVID-19 deaths are due a dysregulated immune system rather than a direct cytopathic effect of the virus [9]. Most of those dying also spend a considerable time (2 to 3 weeks) in hospital before succumbing and it may even be longer from the onset of symptoms [9]. This would mean that they might not actually harbour viable virus at the time of death. While patients who recover from mild to moderate disease are allowed to return to work after 2 weeks of onset of illness, the regulations for management of deceased are still stringent, leaving a question which begs answering: Do the current deceased management regulations which are based on other more fatal and virulent communicable disease need to be revisited with a fresh perspective to allow for a return from this virtually charged “new normal” to the conventional performance of rites [10]?

Human resilience in the face of adversity will ultimately help the bereaved cope with grief. However, an objective method to evaluate the need for adopting innovative modes of support need to be assessed before embracing them as a part of the forbearance.
